# Successful Conversion Surgery for Unresectable Microsatellite Instability–High Pancreatic Tail Cancer Following Pembrolizumab Therapy

**DOI:** 10.70352/scrj.cr.25-0174

**Published:** 2025-07-02

**Authors:** Nobuhiro Harada, Masanao Kurata, Yasuji Seyama, Chikara Shirata, Hiroko Okinaga, Toshihiro Okuya, Mizuka Suzuki, Shinichiro Horiguchi

**Affiliations:** 1 Department of Hepato-Biliary-Pancreatic Surgery, Tokyo Metropolitan Cancer and Infectious Diseases Center Komagome Hospital, Tokyo, Japan; 2 Department of Medical Oncology, Tokyo Metropolitan Cancer and Infectious Diseases Center Komagome Hospital, Tokyo, Japan; 3 Department of Radiology, Tokyo Metropolitan Cancer and Infectious Diseases Center Komagome Hospital, Tokyo, Japan; 4 Department of Pathology, Tokyo Metropolitan Cancer and Infectious Diseases Center Komagome Hospital, Tokyo, Japan

**Keywords:** unresectable pancreatic cancer, pembrolizumab, conversion surgery

## Abstract

**INTRODUCTION:**

Pembrolizumab was approved for the treatment of microsatellite instability (MSI)–high pancreatic cancer that is unresponsive to systemic chemotherapy. However, pancreatic cancer has a low prevalence of MSI-high status (<1%). We report a rare case of MSI-high pancreatic tail cancer successfully treated with pembrolizumab, which enabled conversion surgery to be performed.

**CASE PRESENTATION:**

A 78-year-old male patient received the diagnosis of unresectable metastatic (UR-M) pancreatic tail cancer with para-aortic and anterior mediastinal lymph node metastases. Tests found high MSI due to the loss of MSH2 and MSH6 expression. Following 6 courses of pembrolizumab, he achieved a partial response. Pembrolizumab was discontinued after immune-related renal dysfunction developed during the 7th course. Fluorodeoxyglucose positron emission tomography/computed tomography demonstrated decreased uptake in the anterior mediastinal and para-aortic lymph nodes, allowing a radical resection to be performed. The patient underwent a radical antegrade modular pancreatosplenectomy with a left adrenalectomy and para-aortic lymph node sampling. Currently, at postoperative month 6, he is alive and recurrence-free. The present case is an extremely rare instance of MSI-high, unresectable pancreatic cancer that was removed by curative resection after pembrolizumab therapy.

**CONCLUSIONS:**

The present rare case of conversion surgery for MSI-high UR-M pancreatic tail cancer, achieving a pathological partial response after pembrolizumab treatment, highlights the potential of pembrolizumab against this specific molecular subtype of pancreatic cancer and underscores the clinical significance of proactively testing for MSI to identify candidates for immunotherapy and curative conversion surgery.

## Abbreviations

CA19-9
: cancer antigen 19-9
CEA
: carcinoembryonic antigen
dMMR
: deficient mismatch repair
DUPAN-2
: pancreatic cancer-associated antigen-2
EUS-FNA
: endoscopic ultrasound fine-needle aspiration
FDG-PET
: ^18^F-fluorodeoxyglucose positron emission tomography
MMR
: mismatch repair
MSI
: microsatellite instability
NAC
: neoadjuvant chemotherapy
ORR
: overall response rate
PD-1
: programmed cell death-1
RAMPS
: radical antegrade modular pancreatosplenectomy
RECIST
: response evaluation criteria in solid tumors
SUVmax
: maximum standardized uptake value
UR-M
: unresectable metastatic cancer

## INTRODUCTION

Pembrolizumab, an anti-PD-1 monoclonal antibody, is used as a standard treatment in immunotherapy against various cancers, including MSI-high solid tumors.^1)^ The 2018 KEYNOTE-158 trial demonstrated the efficacy of pembrolizumab against MSI-high solid tumors, leading to its approval for insurance coverage regardless of carcinoma type.^2)^ In February 2019, pembrolizumab was approved for the treatment of MSI-high pancreatic cancer that is unresponsive to systemic chemotherapy. However, pancreatic cancer has a low prevalence of MSI-high status (<1%).^3)^ A recent case report has demonstrated that pembrolizumab may be effective against this rare subgroup.^4)^ Herein, we report a rare case of MSI-high pancreatic tail cancer successfully treated with pembrolizumab, which enabled conversion surgery to be performed.

## CASE PRESENTATION

**Fig. 1:**
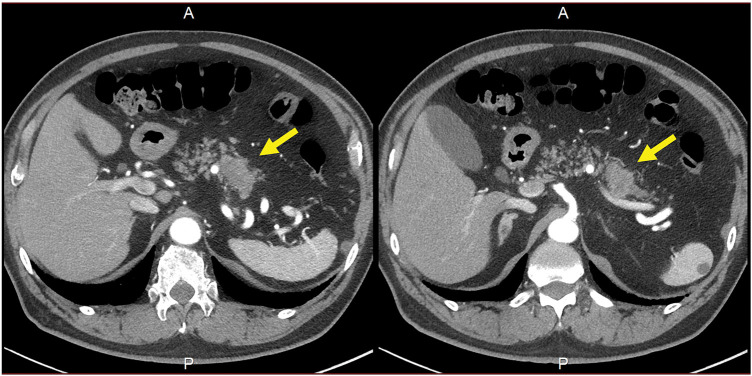
Axial view on contrast-enhanced CT. A 38.6-mm mass in the pancreatic tail can be seen invading the splenic artery (arrows: primary tumor).

**Fig. 2:**
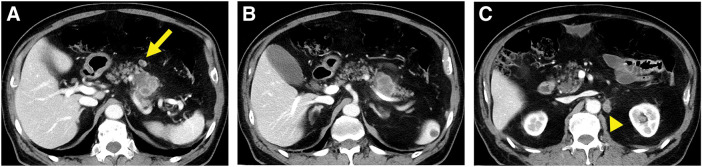
Axial view on contrast-enhanced CT after NAC using gemcitabine plus S-1. (**A**) Lymph node enlargement around the primary tumor (arrow). (**B**) No change in the size of the primary tumor. (**C**) Para-aortic lymph node (13.9 mm) (arrowhead). NAC, neoadjuvant chemotherapy

**Fig. 3:**
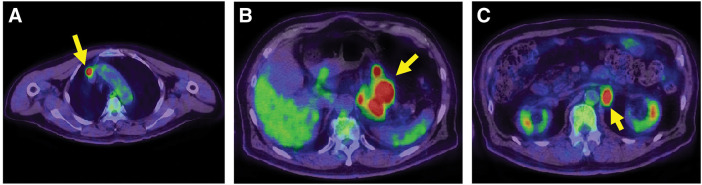
FDG-positron emission-tomography-computed tomography. (**A**) High FDG uptake in the anterior mediastinal lymph node was observed (SUVmax = 16.48) (arrow). (**B**) High FDG uptake in the primary tumor and surrounding lymph nodes was observed (SUVmax = 13.15) (arrow). (**C**) High FDG uptake in the para-aortic lymph node was observed (SUVmax = 9.96) (arrow). FDG, ^18^F-fluorodeoxyglucose; SUVmax, maximum standardized uptake value

**Fig. 4:**
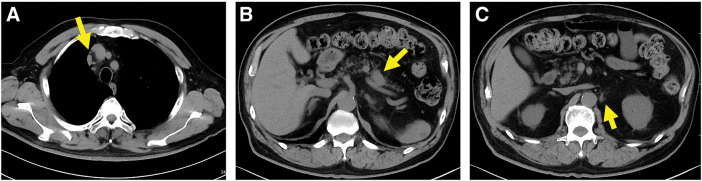
Axial view on computed tomography after 6 courses of pembrolizumab therapy. (**A**) The anterior mediastinal lymph node shrank (3.0 mm) (arrow). (**B**) The primary tumor shrank (26.3 mm) (arrow). (**C**) The para-aortic lymph node shrank (3.0 mm) (arrow).

**Fig. 5:**
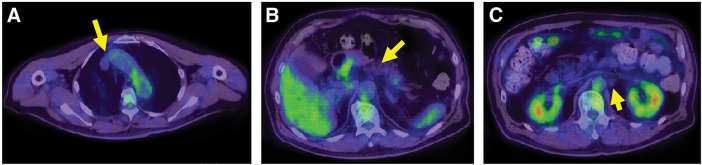
FDG-positron emission-tomography-computed tomography after 6 courses of pembrolizumab therapy. (**A**) FDG accumulation in the anterior mediastinal lymph node resolved (arrow). (**B**) FDG accumulation in the primary tumor and surrounding lymph nodes decreased (SUVmax = 1.65) (arrow). (**C**) FDG accumulation in the para-aortic lymph node resolved (arrow). FDG, ^18^F-fluorodeoxyglucose; SUVmax, maximum standardized uptake value

**Fig. 6:**
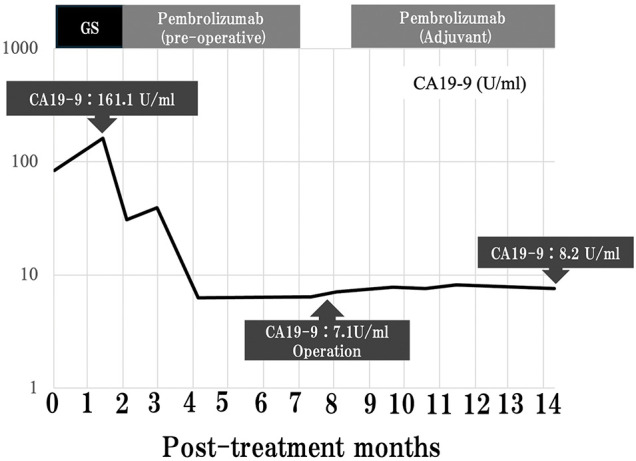
Changes in tumor markers over time. CA19-9, cancer antigen 19-9; GS, gemcitabine plus S-1

**Fig. 7:**
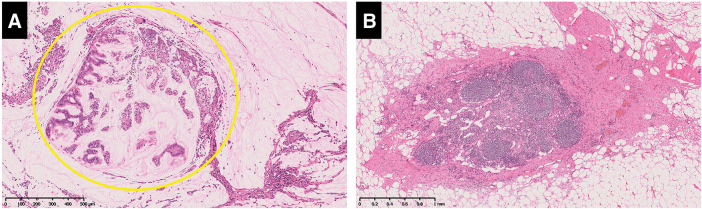
Histopathological findings (hematoxylin–eosin staining). (**A**) A few viable tumor cells were suspended in the mucin of the primary pancreatic tumor (yellow circle). (**B**) An atrophied lymph node with some scarring can be seen. No tumor cells were found in the para-aortic lymph node. Histopathological diagnosis : Pt, TS2, size 20 × 14 mm, mucinous carcinoma, nodular type, ypT3, int, INFb, ly0, V0, Pn0, mpd0, pS1, pPR1, pPV0, pA0, pPL0, pOO0, pPCM0, pDPM0, n(0/23), and Evans classification grade IIb.

A 78-year-old male patient with a history of right nephrectomy for right renal clear cell carcinoma presented for follow-up. CT revealed a hypovascular mass in the pancreatic tail (**Fig. 1**). EUS-FNA confirmed the presence of adenocarcinoma. Initially diagnosed as resectable pancreatic tail cancer (cT3N1M0 cStageIIB), the lesion was treated with 2 courses of NAC consisting of gemcitabine plus S-1. However, follow-up CT post-NAC therapy revealed lymph node enlargement around the primary tumor and in the para-aortic region (**Fig. 2**). EUS-FNA of the para-aortic lymph node found an adenocarcinoma metastasis. FDG-PET/CT demonstrated increased uptake in the primary tumor (SUVmax 16.48), para-aortic lymph node (SUVmax 13.15), and anterior mediastinal lymph node (SUVmax 9.96), indicating UR-M pancreatic tail cancer (**Fig. 3**). The patient had a history of surgery for sigmoid colon cancer at the age of 42 years and endoscopic submucosal dissection for transverse colon cancer at the age of 73 years. Furthermore, his younger brother, nephew, and niece had colon cancer, fulfilling Amsterdam Criteria II. Based on the medical and family history, Lynch syndrome was strongly suspected. An MSI evaluation of an EUS-FNA specimen of the pancreas revealed loss of expression of MMR proteins, MSH2 and MSH6. Based on the MSI-high status, pembrolizumab monotherapy was begun as 2nd-line treatment. After 6 courses, follow-up CT (**Fig. 4**) found significant tumor shrinkage; the primary tumor, para-aortic lymph node, and the anterior mediastinal lymph node had shrunk from 38.6 to 26.3, 13.9 to 3.0, and 10 to 3.0 mm, respectively. The 7th pembrolizumab course was discontinued after suspected immune-related renal dysfunction developed. The RECIST confirmed a partial response with a 40% reduction in tumor size. Subsequent FDG-PET/CT (**Fig. 5**) demonstrated decreased FDG uptake in the primary lesion (SUVmax 1.65) and a cessation of uptake in both the mediastinal and para-aortic lymph nodes, indicating reduced tumor viability. Tumor markers, CEA, CA19-9, and DUPAN-2, also normalized after pembrolizumab therapy (**Tables 1**, **2** and **Fig. 6**). Based on these findings, the diagnosis was revised to ycT3N0M0 ycStage IIA, and conversion surgery was deemed feasible. RAMPS, including left adrenal gland resection and para-aortic lymph node sampling, was performed. Intraoperative washing cytology was negative. Intraoperative frozen section diagnosis of the para-aortic node after curative resection was also negative. The operative time was 350 min, and the amount of blood loss was 508 mL. There were no postoperative complications, and the patient was discharged on postoperative day 11. The histopathological diagnosis was 20 × 14 mm Pt, TS2, mucinous carcinoma, nodular type, ypT3, pS1, pPR1, pPCM0, pDPM0, n(0/23), and Evans classification grade IIb. No tumor cells were found in the para-aortic lymph node (**Fig. 7**). Postoperative adjuvant pembrolizumab was administered for 6 months, and the patient is currently alive and recurrence-free.

**Table 1:** Laboratory data on preoperative

	Value	Unit	Reference range
WBC	5.6	×10^3^/μL	3.3–8.6
Hb	9.7	g/dL	13.7–16.8
Plt	15	×10^4^/μL	15.8–34.8
Alb	3.5	g/dL	4.1–5.1
AMY	74	U/L	44–132
AST	15	U/L	13–30
ALT	11	U/L	10–42
LDH	175	U/L	124–222
γGTP	15	U/L	13–64
BUN	26	mg/dL	8.0–20.0
CRE	1.87	mg/dL	0.65–1.07
Na	140	mEq/L	138–145
K	4.5	mEq/L	3.6–4.8
Cl	109	mEq/L	101–108
T-Bil	0.4	mg/dL	0.4–1.5
CRP	0.07	mg/dL	0.00–0.14
CEA	3.6	ng/mL	0.0–5.0
CA19-9	7.1	U/ml	0.0–37.0
DUPAN2	<25	U/ml	0–150
Span-1	6.5	U/mL	0–30.0
Elastase1	<80	ng/mL	<300

γGTP, γ-glutamyl transpeptidase; Alb, albumin; ALT, alanine aminotransferase; AMY, amylase; AST, asparate aminotransferase; BUN, blood urea nitrogen; CA19-9, cancer antigen 19-9; CEA, carcinoembryonic antigen; Cl, chlorine; CRE, creatinine; CRP, C-reactive protein; DUPAN2, duodenal pancreatic cancer associated antigen-2; Hb, hemoglobin; K, potassium; LDH, lactate dehydrogenase; Na, sodium; Plt, platelets; T-Bil, total bilirubin; WBC, white blood cells

**Table 2:** Trend of tumor marker

	Value					
	Pretreatment	Progression	Preoperative	Postoperative	Unit	Reference range
CEA	3.8	6.9	3.6	3.3	ng/mL	0.0–5.0
CA19-9	83.5	161.1	7.1	8.2	U/mL	0.0–37.0
DUPAN2	<25	<25	<25	<25	U/mL	0–150
Span-1	27	34	6.5	5.9	U/mL	0–30.0

CA19-9, cancer antigen 19-9; CEA, carcinoembryonic antigen; DUPAN2, duodenal pancreatic cancer associated antigen-2

## DISCUSSION

The present case report described a rare instance of successful conversion surgery following pembrolizumab therapy, achieving a pathological partial response in a patient with unresectable advanced MSI-high pancreatic cancer.

Pembrolizumab, an immune checkpoint inhibitor commonly used in cancer immunotherapy, has been attracting attention in recent years. It is a humanized monoclonal antibody binding to PD-1 on T cells, thereby reactivating the T cells and causing antitumor effects. The latter are particularly potent against MSI-high solid tumors, which contain numerous gene mutations.^1)^ MMR protein (MLH1, MSH2, MSH6, and PMS2) expression in tumor tissues was examined immunohistochemically to assess for dMMR, defined as the loss of expression of 1 or more proteins. In the present case, the loss of MSH2 and MSH6 expression led to the diagnosis of MSI-high status.^5)^ The frequency of dMMR in pancreatic cancer is around 1%,^6–9)^ and data on the pancreatic cancer cohort in KEYNOTE-158 disappointingly demonstrated an ORR of pembrolizumab <20% in this subgroup.^2)^ On the other hand, Coston et al. reported an ORR of pembrolizumab of 75% (20% complete response in retrospective data).^10)^

There are limited reports of conversion surgery after pembrolizumab for unresectable pancreatic cancer. Ito et al. have reported a case of conversion surgery with excellent efficacy of pembrolizumab in a patient with unresectable pancreatic cancer with para-aortic lymph node metastasis.^4)^ In that report, pembrolizumab was administered for 19 courses as a 4th line after gemcitabine plus nab-paclitaxel, modified oxaliplatin plus irinotecan plus levofolinate plus fluorouracil, and albumin-suspended irinotecan plus levofolinate plus fluorouracil, and histopathological findings revealed that the Evans classification of the primary tumor was grade IV, and there was viable tumor cell metastasis in the para-aortic lymph node. In our case, because we strongly suspected Lynch syndrome based on the medical history and family history, we were able to perform MSI evaluation before 2nd-line treatment, and early curative treatment was performed after 6 courses of pembrolizumab. As for histopathological findings, it was revealed that the Evans classification of the primary tumor was grade IIb, and there were no viable tumor cells in the para-aortic lymph node where biopsy had detected cancer cells. To the best of our knowledge, this is the 2nd report of successful conversion surgery for MSI-high UR-M pancreatic cancer following pembrolizumab. The present case demonstrated a remarkable response to pembrolizumab, which enabled a curative resection to be performed. Although MSI evaluation has problems with appropriate tissue collection and timing, the present findings underscore the importance of MSI testing in pancreatic cancer to identify candidates for immunotherapy.

Lynch syndrome should be suspected if MSI-high status is found. The family history of our patient, which included cancers fulfilling Amsterdam Criteria II and the revised Bethesda Guidelines, raised the index of suspicion for Lynch syndrome.^11)^ While the patient’s family declined the recommendation for genetic testing, the findings of the case emphasize the importance of genetic counseling and testing whenever Lynch syndrome is suspected in the context of MSI-high pancreatic cancer. Following the administration of NAC-GS consisting of a gemcitabine plus S-1 regimen, the patient's condition progressed to unresectable pancreatic tail cancer with a distant metastasis. Given the limited efficacy of both gemcitabine and fluoropyrimidine-based regimens, alternative therapeutic approaches were considered. The patient’s personal and family history, and the suspicion of Lynch syndrome, prompted MSI testing, which revealed MSI-high status. In a previous report, MSI evaluation was performed after 3rd-line chemotherapy for UR pancreatic cancer, and pembrolizumab was introduced.^4)^ This crucial finding led to an earlier choice of pembrolizumab as the 2nd-line therapy.

Timing of conversion surgery for UR-M pancreatic cancer remains controversial. According to the 2022 Japanese Pancreatic Treatment Guidelines,^12)^ evidence for surgery following multidisciplinary intervention for UR-M pancreatic cancer is limited. However, in the present case, the decision to perform a curative resection was prompted by the development of immune-related adverse events (renal dysfunction), the significant tumor response observed on imaging studies, and the marked decrease in tumor markers. The FDG-PET/CT findings, which demonstrated decreased tumor viability in both the primary lesion and metastases, were particularly crucial in determining the feasibility of resection. While postoperative chemotherapy for conversion surgery in UR-M pancreatic cancer lacks strong supporting evidence,^13–15)^ adjuvant pembrolizumab was administered for 6 months on the basis of the current evidence for resectable pancreatic cancer^16)^ and the patient’s excellent response to preoperative pembrolizumab administration. Although the renal dysfunction required the 7th course of pembrolizumab therapy to be discontinued, postoperatively the condition improved enough to allow adjuvant pembrolizumab administration. We believe that removal of the mediastinal lymph nodes is necessary to achieve a cancer-free state. Considering the surgical invasiveness, we initially considered a 2-stage operation (1st surgery: primary tumor resection, 2nd surgery: mediastinal lymph node resection). The pathological diagnosis of the para-aortic lymph node was negative for cancer. Because the metastatic lesion has become so small, it may be difficult to find the target lymph node using minimally invasive procedures such as mediastinoscopy, and more invasive open-chest surgery may be necessary for its removal. In addition, pembrolizumab is being administered as postoperative adjuvant therapy. Based on the above, we decided to consider lymph node dissection only if the mediastinal lymph node increased in size. Further research is needed to define the optimal treatment strategy and to identify biomarkers that are predictive of the response to immunotherapy in patients with advanced MSI-high pancreatic cancer.

## CONCLUSIONS

The present rare case of conversion surgery for MSI-high UR-M pancreatic tail cancer, achieving a pathological partial response after pembrolizumab treatment, highlights the potential of pembrolizumab against this specific molecular subtype of pancreatic cancer and underscores the clinical significance of proactively testing for MSI to identify candidates for immunotherapy and curative conversion surgery.

## ACKNOWLEDGMENTS

We would like to thank Mr. James R. Valera for the English language editing.

## DECLARATIONS

### Funding

All funding sources for this study have nothing to report.

### Authors’ contributions

NH was the chief physician of this patient and was in charge of surgery.

NH collected data and drafted the article.

MK and YS helped NH to revise the article.

All authors were engaged in the medical treatment of this patient together and approved the final manuscript.

All authors read and approved the final manuscript.

### Availability of data and materials

The authors confirm that the data supporting the findings of this study are available within the article.

### Ethics approval and consent to participate

This work does not require ethical considerations or approval. Informed consent to participate in this study was obtained from the patient.

### Consent for publication

Informed consent for publication of this case report was obtained from the patient.

### Competing interests

The authors have no competing interests to disclose.
